# The long-term effects of reproductive health education among primary and secondary school students: a longitudinal quasi-experimental study in rural Tanzania

**DOI:** 10.1186/s12978-023-01662-4

**Published:** 2023-08-29

**Authors:** Yoko Shimpuku, Naoki Hirose, Sanmei Chen, Dorkasi L. Mwakawanga, Niko Madeni, Frida Madeni, Mariko Komada, Ayaka Teshima, Mayu Morishima, Yasunobu Ando, Koji Takahama, Atsushi Nishida

**Affiliations:** 1 Graduate School of Biomedical and Health Sciences, Hiroshima University, 1-2-3 Kasumi, Minami-Ku, Hiroshima, 734-8553 Japan; 2 School of Nursing, Muhimbili University of Health and Allied Sciences, Dar es Salaam, Tanzania; 3 Magunga Hospital, Korogwe, Tanga, Tanzania; 4 The New Rural Children Foundation, Dar es Salaam, Tanzania; 5 NPO Class for Everyone, Kanagawa, Japan; 6 Department of Psychiatry and Behavioral Science, Tokyo Metropolitan Institute of Medical Science, Tokyo, Japan

**Keywords:** Adolescent, Reproductive health, Adolescent pregnancy, Health Education, Tanzania, Quasi-experimental school study

## Abstract

**Background:**

Adolescent pregnancy remains a major global health issue, increasing the risk of complications during pregnancy and childbirth in mothers and babies. In Tanzania, adolescent pregnancy threatens girls’ education and makes it difficult for them to obtain a proper job; hence, the majority fall into poverty. Previous studies have developed and conducted reproductive health education for adolescent students; however, they evaluated only the effect immediately after education. Therefore, this study investigated the effects of reproductive health education on attitudes and behaviors toward reproductive health among adolescent girls and boys one year after the intervention in rural Tanzania.

**Methods:**

A longitudinal quasi-experimental study was conducted with 3295 primary and secondary students (2123 in the intervention group, 1172 in the control group) from three purposefully selected wards in Korogwe District. In the intervention group, the students received reproductive health education. We used paper-based questionnaires to evaluate the effect of the adolescent education program on attitudes and behaviors toward reproductive health education. To analyze the association between the intervention and each outcome, mixed-effect multiple regression analyses was conducted.

**Results:**

The mean age, primary school proportion, and female proportion of the intervention and the control group was 13.05 (standard deviation (SD) 1.59), 14.14 (SD 1.7), 77.9% and 34.3%, and 54.2% and 52.6%, respectively. There was no statistically significant effect of reproductive health education on adolescent health attitudes and behaviors in the multiple regression analyses (coefficient: − 0.24 (95% confidence interval (CI): − 0.98 to 0.50), coefficient: 0.01 (95%CI: − 0.42 to 0.43)).

**Conclusion:**

A statistically significant effect of reproductive health education on adolescent health attitudes and behaviors was not found. An effective reproductive health education intervention to improve the attitude and behaviors of reproductive health among Tanzania adolescents in the long term remain to be determined, particularly in real-world settings.

*Trial registration* The National Institute for Medical Research, Tanzania (NIMR/HQ/R.8a/Vol. IX988).

## Plain Language Summary

Adolescent pregnancy increases the risk of complications during pregnancy and childbirth, which could cause death among 15–19-year-old girls. In Tanzania, one in four adolescents aged 15–19 began childbearing. However, there is no officially recognized curriculum for reproductive health in schools. Additionally, cultural and traditional norms prevent parents from discussing sexuality with their children. A solution to this issue is for a third party, such as a non-profit organization, to provide adolescents with adequate reproductive health education in schools. Previous studies have developed and evaluated a reproductive health education program and found significant effects on improved knowledge and behavior among adolescent girls and boys immediately after the intervention. This study examined the effects of reproductive health education on adolescents` attitudes and behavior toward reproductive health one year after the intervention in rural Tanzania. We could not find a statistically significant effect of reproductive health education on adolescents’ attitudes and behavior during a one-year period in the multiple regression analyses. These results could have been influenced by the location of the intervention, contents, period of evaluation, and other potentially unknown factors.

## Background

Adolescent pregnancy is a major global health issue that contributes to maternal and newborn mortality. It is estimated that 21 million girls aged 15–19 years become pregnant, 12 million give birth, and 777,000 girls give birth below the age of 15 each year in low- and middle-income countries [1–3]. According to the world health organization (WHO), adolescent pregnancy is defined as “pregnancy in a woman aged 10–19 years [4]. Pregnancy during adolescence increases the risk of complications during pregnancy and childbirth such as eclampsia, puerperal endometritis, and infection, which are the leading causes of death in 15–19-year-old girls [5]. Moreover, in the developing world, at least 10 million unintended pregnancies occur annually [1]. This could lead to unsafe induced abortion, which is another cause of maternal death, with an estimated 6,230,000 deaths, the highest occurring in East Africa [6]. Additionally, adolescent pregnancy increases the risk of stillbirth, low birth weight, and newborn deaths [4].

In Tanzania, a significant number of adolescent girls become sexually active at the age of 15, and 60% of women have had sex before the age of 18 [7]. By 2016, one in four adolescents aged 15–19 began childbearing [8]. The adolescent fertility rate increased from 116 to 132 between 2010 and 2015/16, the 17th highest in Africa [9]. Until the recent policy change, pregnant girls attending school were compelled to withdraw from school between 2003 and 2011 in Tanzania, and 55,000 children were withdrawn from school due to pregnancy [9]. Dropping out from school makes it difficult for girls to get a job, and consequently, many fall into a cycle of multigenerational poverty. Thus, interventions for reducing unintended adolescent pregnancies are of substantial benefit to adolescents, their following generations, and society as a whole.

A systematic review and meta-analysis that was conducted among 254,350 participants to estimate the prevalence and sociodemographic determinant factors of adolescent pregnancy in African countries identified several factors such as residing in rural areas, being married, not attending school, having no maternal education, lack of father's education, and lack of parent-to-adolescent communication on sexual and reproductive health (SRH) issues [10]. Another study conducted by McCleary-Sills et al., focused on gendered norms, sexual exploitation and adolescent pregnancy reported four major risk factors that undermined girls' ability to protect their health and well-being. These include poverty that pushed them into having sex to meet basic needs, sexual expectations of older men and boys, age, rape, and coercive sex (including sexual abuse from an early age), and unintended pregnancy [11]. Prevention of unwanted pregnancy needs collaborative efforts, and healthcare providers need knowledge about reproductive health rights for girls and boys and should adhere to this by providing youth-friendly reproductive healthcare. While girls require proper knowledge about pregnancy and its consequences, boys need to know what issues girls face when they unintentionally become pregnant [12]. A study conducted in Tanzania showed that personal, cultural, and religious lenses affected healthcare professionals’ decisions about whether to provide contraception to adolescent girls [13]. The latter serve as potential targets for intervention to empower adolescents’ knowledge in contexts where adolescents have limited access to sexual and reproductive health training.

To the best of our knowledge, there are no sex education classes in Tanzania, and life skills teachers often lack special training on sexual and reproductive health [14, 15]. Sexual and reproductive health education in school curriculum is integrated in biology subject for secondary schools and in civics and morals and science and technology subjects for primary schools, and not as stand-alone subject [16]. Moreover, “abstinence-only” sex education, HIV/AIDS, puberty, STIs and gender-based violence is often the content taught at school and provides Tanzanian adolescents with insufficient information on how to prevent unwanted pregnancy [17]. Even at home, cultural norms and traditional values make it difficult for parents and children to discuss sexuality [18]. Several studies in Africa have reported that parents hesitated to talk about sexuality with their children because of feelings of inadequate knowledge [19–21]. Discussions between parents and their children also turned out to be not knowledge-providing but prohibitive and one-sided [22, 23]. Therefore, there is an urgent need for third parties to provide proper and adequate knowledge and comprehensive sexuality education messages [24, 25].

In response to this need, Madeni et al., developed and conducted a reproductive health education awareness program in Tanzania using a picture drama of a girl who experienced adolescent pregnancy [26]. The study showed significant beneficial effects of this program on adolescent boys’ and girls’ knowledge, attitudes, and reproductive health behavior. A subsequent study in rural Tanzania showed less successful results with the same intervention and measurements [27]. In both studies, the authors discussed the difference in responses to the program due to lower levels of educational attainment and culture in rural villages and concluded that revisions must be made to the existing program for adolescents living in rural areas, by considering their specific culture, practices, and learning needs. In addition, both studies measured the learning effects immediately after the program. Therefore, the long-term effect, that is, the effect after a year, needs to be analyzed.

As a next step, the Japan international cooperation agency (JICA) Partnership Project, “Adolescent education project to prevent social isolation due to unwanted pregnancy and school dropouts,” was launched in rural Tanzania in 2017 by a Japanese Non-Profit Organization (NPO), Class for Everyone, in collaboration with a local Non-Governmental Organization (NGO), the New Rural Children Foundation. To empower students to actively participate in the program, the project added role-play in their teaching and asked students how they felt when they played roles in the story and how they could have behaved to prevent unwanted pregnancy, aiming to use more experiential learning to address the rural culture. They included more open discussions among students under the guidance of a third party, the local NGO. This project followed up with the students for one year and evaluated the differences in reproductive attitudes, behavior, and logical thinking. Therefore, in this quasi-experimental study, the aim was to investigate the effects of reproductive health education on the attitudes and behavior toward reproductive health among adolescent girls and boys in rural Tanzania.

## Methods

### Study design

A longitudinal quasi-experimental study.

#### Hypotheses

There is a significant increase in the positive attitudes and behavior concerning reproductive health among adolescent girls and boys through the intervention of reproductive health education.

### Study setting

Data collection took place in 43 primary schools (14 control and 29 intervention) and 15 secondary schools (9 control and 6 intervention) in three wards of Korogwe District, Tanzania. The number of schools were all that were introduced by the district council. The wards were selected based on their location as very rural, such as village areas, or town and the other as middle. All schools within the wards were included in the study. Korogwe is located between two major cities, Dar es Salaam and Arusha. The total area is 3203 km^2^, and the total population is 242,038 with 76 km^2^ density [28]. Korogwe is best known for its national park with natural reserves. There was one hospital, three health centers, and 48 dispensaries. The project was developed by a Japanese NPO Class for Everyone and was implemented in collaboration with the local NGO New Rural Children Foundation. The site was selected because of the following reasons: (1) The site was considered rural compared to major cities, (2) The local NGO could run the program with the support of the Japanese NPO, and (3) The school principals and district education officers were supportive of the program.

### Study population and enrollment

**Fig. 1:**
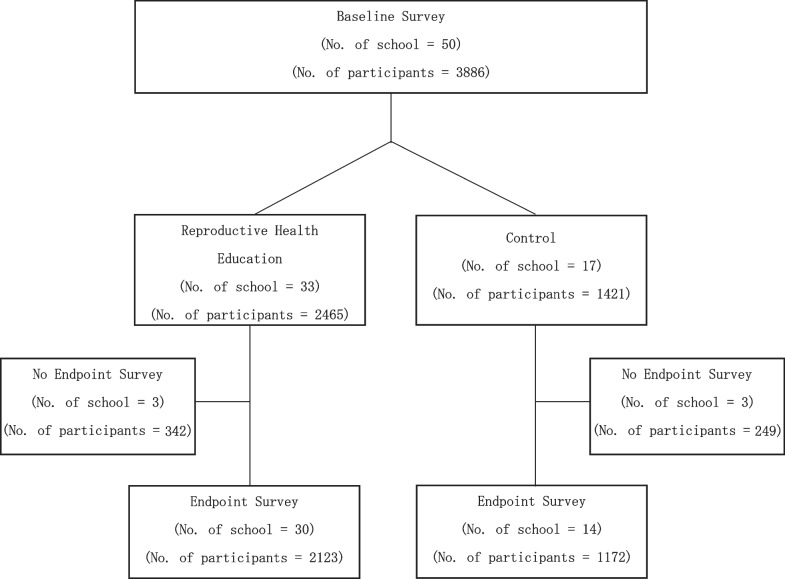
The process of recruitment

The target group for this study was students in standard 5 and 6 in primary schools and form 1 and 2 in ordinary secondary schools. As a follow-up was planned one year after the baseline survey, the last year of both schools was intentionally avoided (standard 7 in primary schools and form 3 and 4 in secondary school) because students would graduate, and it would be difficult to follow them up. The ages of those grades varied because students start primary and secondary schools at non-specific ages in Tanzania, ranging from six to ten years. From the list of all schools in the three wards, the schools were assigned to either the intervention or control group based on geographical information, such as rural or town and the other as middle so that both groups had schools in comparative locations. The study setting is generally rural, but some places were closer to shops and main road, compared to others living in mountain areas, and therefore, we technically called them town or rural according to the bureaucratic categorization by the council. Random assignment or assigning the same number of schools to each group could not be applied because it was a JICA project that required the project to provide education to the maximum number of schools. The recruitment process is illustrated in Fig. 1.

The project team explained the project to the head teachers, and they were asked to set up a classroom for students to participate. The project team explained students the purpose and content of the education and research.

### Sampling

The project team conducted a meeting with all school principals in the three wards to explain the project and to seek cooperation. With permission from the local council, the team visited the schools and explained the project to the teachers. The teachers cooperated in the classroom, and the team explained the project and data collection to students. As this study was conducted as an education project in classrooms, the team included all students who (1) were in the grades described above and (2) agreed to participate in the study. Students who were absent from class on the education day were excluded. From the local NGO, the team expected to include approximately 3000 students in the project.

### Intervention

In the intervention group, the students received reproductive health education. The reproductive health education was originally developed by Madeni et al. [24] as a drama story, and later, picture books were printed for each student to receive and take it home. The story was about an adolescent girl who unexpectedly became pregnant. She stopped going to school because of physical symptoms, including nausea and vomiting. The boyfriend who impregnated her denied his responsibility and left her behind. Her parents later got to know about her pregnancy and excluded her from their house because of the social stigma. After reading the book, students were asked questions about the contents, such as why this issue happened to the girl, how it could be prevented, and how the boy should have acted. Students were then asked to act in role-play as they discussed how to prevent unwanted pregnancy. The questionnaire was administered before initiating the project and after the education project’s completion at one year later. The control group received regular education and completed the survey at the start and at the end of the project. The program took approximately 90 min and one time; however, students kept the book at hand and were able to read it as they wished.

### Procedures

Paper-based questionnaires were used for the data collection. The data included demographic information, attitudes and behavior regarding reproductive health, and logical thinking. The background data included sex, age, grade, religion, ethnic group, number of siblings, plan after graduation, menstruation or bed wetness, experience of sex, experience of forced sex, talking with parents about pregnancy or sexual issues, and talking with parents about everyday life. The outcome of this study was measured using the questionnaire on attitudes and behaviors in reproductive health in Swahili. The questionnaire on attitudes and behaviors in reproductive health was originally developed by Madeni et al., to evaluate the effect of the adolescent education program, which was used as part of this study [26]. The attitude test consists of seven items rated on a 5-point Likert scale (1 = strongly disagree; 5 = strongly agree). The possible scores range from 7 to 35. A high score indicates that they can escape from situations that put them in danger of unwanted pregnancy. The behavioral test consisted of six items rated on a 5-point Likert scale (1 = strongly disagree; 5 = strongly agree). The possible scores ranged from 6 to 30. A high score indicates good decision-making for saying no to sexual behavior.

### Statistical analyses

The participants’ characteristics were compared between those with and without the intervention. Chi-square test for categorical variables and t-test for continuous variables was used. To analyze the association between the intervention and each outcome, a mixed-effect multiple regression analysis was conducted, where school was included as a random effect. The outcomes of these analyses were differences between the reproductive health attitude and behavior scores at 12 months follow-up and baseline. The primary analysis cohort was the overall cohort. In additional analyses, we conducted several mixed-effect multiple regression analyses for cohorts stratified by age (low: ≤ 13; high > 13), the experience of menstruation or nocturnal emission (yes or no), and living place (town or rural), considering the possibility of effect modification by these factors. The age was categorized into two groups by a median. All tests were two-tailed, and the threshold of significance was a p-value of < 0.05. All statistical analyses were conducted with R, Version 4.2.1, and Oracle^®^ R Enterprise, Version 1.4.1 (Oracle, Redwood Shores, CA, USA).

## Results

### Socio-demographic information of study participants

In total, the data from 3295 students (2123 in the intervention group and 1172 in the control group) were included in the analysis of reproductive health education. The demographic information from the analyses is shown in Table 1. Among those who received reproductive health education, their mean age was 13.05 (SD1.59). The majority were primary school students (77.9%), living in town (61.5%), Muslims (61.8%), and had brothers or sisters (89.0%). The female-to-male ratio was approximately 50% (54.2% and 45.8%, respectively). At the beginning of the study, the majority reported that they had not started menstruation or nocturnal emissions (71.2%), experienced no sexual intercourse (98.5%), and had a marriage plan in the future (73.4%). The majority responded that they did not talk about sex or pregnancy with their parents (60.7%) and that they had daily conversations with their parents (83.2%). There were differences between the two groups. The mean student age was higher in the control group (p < 0.001). The control group had fewer primary school students and more living in towns (p < 0.001). Further, more control students had started menstruation or nocturnal emissions (p < 0.001), had a future marriage plan (p < 0.001) and talked about sex or pregnancy with their parents (p < 0.001).

**Table 1:** Background information and scores of the measurements

	Overall	Reproductive health education		
		No	Yes	P-value
n	3295	1172	2123	
School				< 0.001
Primary school	2056 (62.4)	402 (34.3)	1654 (77.9)	
Secondary school	1239 (37.6)	770 (65.7%)	469 (22.1)	
Age				< 0.001
Low	1737 (52.7)	368 (31.4)	1369 (64.5)	
High	1537 (46.6)	796 (67.9)	741 (34.9)	
NA	21 (0.6)	8 (0.7)	13 (0.6)	
Age in continuous variable (mean (SD))	13.44 (1.73)	14.14 (1.74)	13.05 (1.59)	< 0.001
Sex				0.527
Female	1767 (53.6)	617 (52.6)	1150 (54.2)	
Male	1527 (46.3)	555 (47.4)	972 (45.8)	
NA	1 (0.0)	0 (0.0)	1 (0.0)	
Living place				< 0.001
In rural	1147 (34.8)	330 (28.2)	817 (38.5)	
In town	2148 (65.2)	842 (71.8)	1306 (61.5)	
Religion				0.015
Christian	1262 (38.3)	481 (41.0)	781 (36.8)	
Muslim	1990 (60.4)	679 (57.9)	1311 (61.8)	
Others	21 (0.6)	9 (0.8)	12 (0.6)	
NA	22 (0.7)	3 (0.3)	19 (0.9)	
Have brother or sister				0.021
No	381 (11.6)	160 (13.7)	221 (10.4)	
Yes	2894 (87.8)	1005 (85.8)	1889 (89.0)	
NA	20 (0.6)	7 (0.6)	13 (0.6)	
Experience of menstruation or nocturnal emission				< 0.001
No	2164 (65.7)	653 (55.7)	1511 (71.2)	
Yes	1102 (33.4)	517 (44.1)	585 (27.6)	
NA	29 (0.9)	2 (0.2)	27 (1.3)	
Experience of sexual intercouse				< 0.001
No	3248 (98.6)	1157 (98.7)	2091 (98.5)	
Yes	18 (0.5)	13 (1.1)	5 (0.2)	
NA	29 (0.9)	2 (0.2)	27 (1.3)	
Marriage plan in the future				< 0.001
No	726 (22.0)	184 (15.7)	542 (25.5)	
Yes	2540 (77.1)	982 (83.8)	1558 (73.4)	
NA	29 (0.9)	6 (0.5)	23 (1.1)	
Talk about sex or pregnancy with parents				< 0.001
No	1899 (57.6)	610 (52.0)	1289 (60.7)	
Yes	1378 (41.8)	560 (47.8)	818 (38.5)	
NA	18 (0.5)	2 (0.2)	16 (0.8)	
Have daily talk with parents				0.086
No	515 (15.6)	173 (14.8)	342 (16.1)	
Yes	2764 (83.9)	997 (85.1)	1767 (83.2)	
NA	16 (0.5)	2 (0.2)	14 (0.7)	
The attitudes of reproductive health score at baseline (mean (SD))	18.43 (5.06)	17.95 (5.06)	18.70 (5.04)	< 0.001
The behaviors of reproductive health score at baseline (mean (SD))	14.62 (3.30)	14.20 (3.05)	14.85 (3.42)	< 0.001
The hope for the future at baseline (mean (SD))	38.89 (4.82)	39.25 (4.72)	38.68 (4.86)	0.002
The attitudes of reproductive health score at final (mean (SD))	17.76 (5.07)	17.66 (5.05)	17.82 (5.09)	0.444
The behaviors of reproductive health score at final (mean (SD))	14.41 (3.06)	14.05 (2.89)	14.61 (3.13)	< 0.001
The hope for the future at final (mean (SD))	38.24 (5.03)	38.99 (4.99)	37.83 (5.01)	< 0.001

**Fig. 2:**
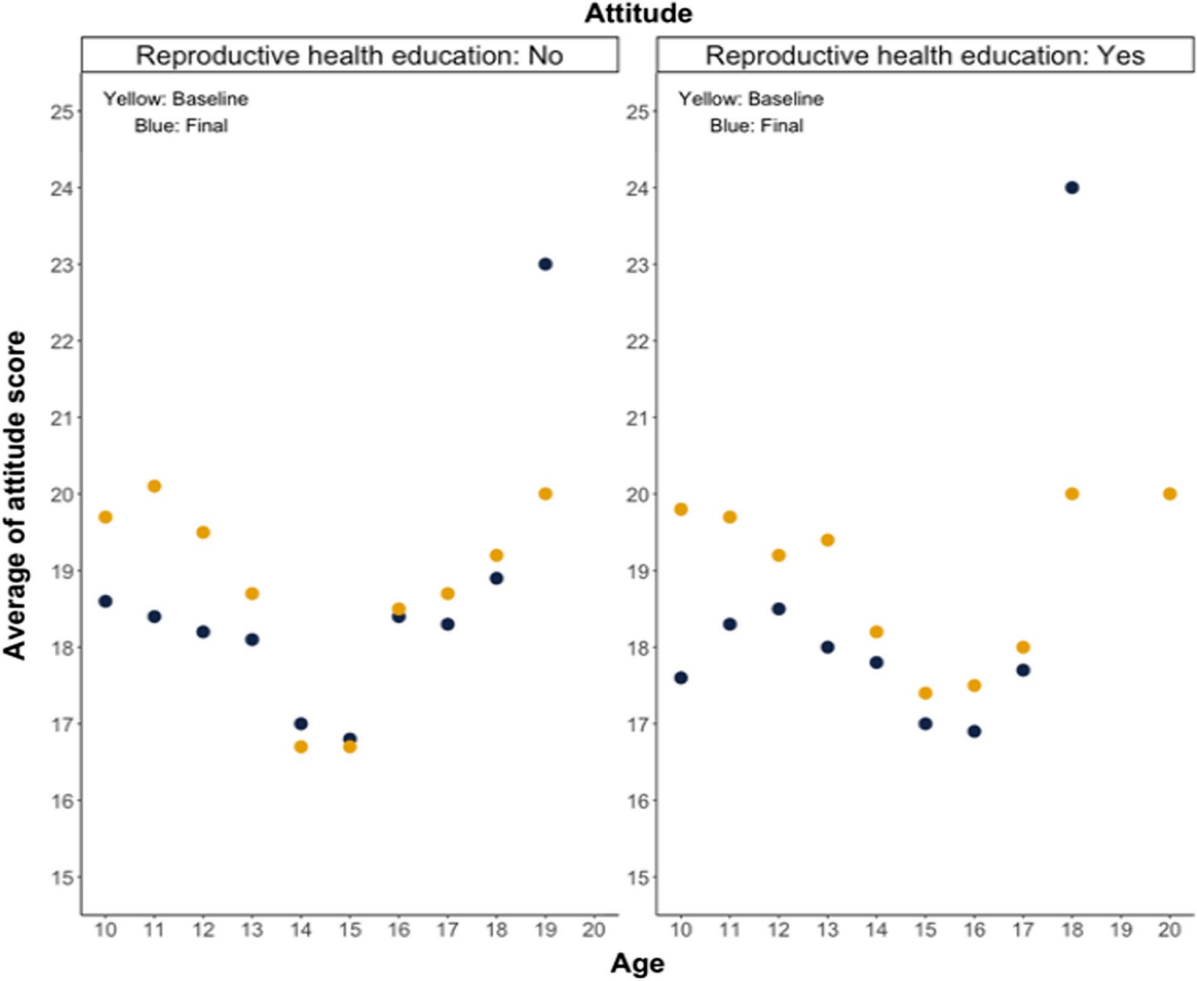
Students’ attitude to reproductive health by age

**Fig. 3:**
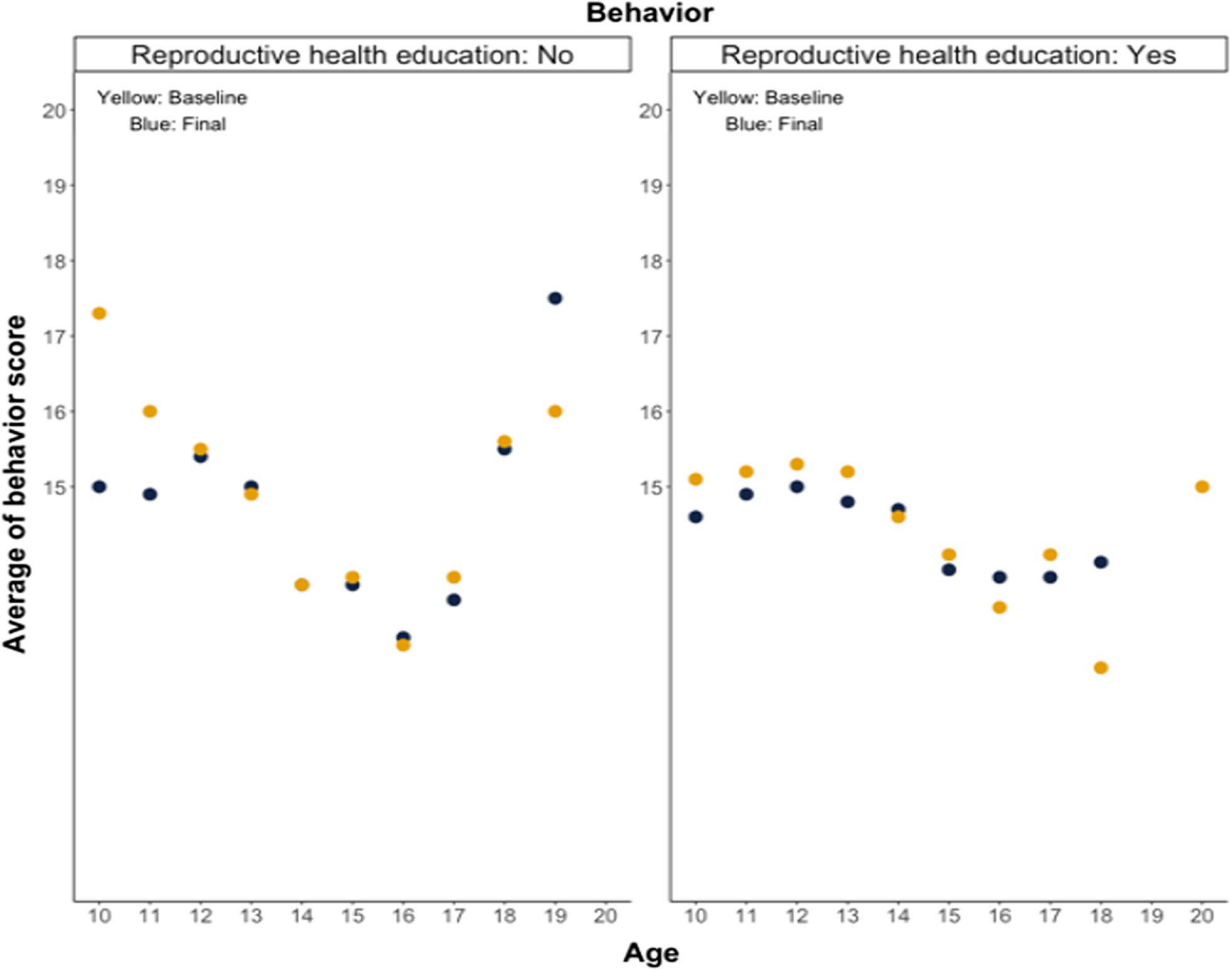
Students’ behavior concerning reproductive health by age

The scores were influenced by age, and Figs. 2, 3 illustrate how reproductive health attitude scores and behavior scores were distributed and changed between groups over time. The description below did not indicate statistical significance, but rather the movement of the scores. Regarding attitude to reproductive health (Fig. 2), the scores decreased until the age of 15–17.5, and the data tended to be lower during the final data collection period than at the baseline. The scores increased after the age of 15–17.5, and the final score also improved. Regarding reproductive health behavior (Fig. 3), the scores decreased until the age of 16 in general. The scores increased after the age of 16, thereby improving the final score.

### Multiple regression analysis

There was no statistically significant difference in the scores of reproductive health attitudes and behavior between the intervention and control groups with respect to reproductive health education (Table 2).

**Table 2:** Multiple regression on reproductive health attitude and behavior

	Coeficience	95% CI		Pr( >|t|)	Coeficience	95% CI		Pr( >|t|)
	Reproductive health attitude				Reproductive health behavior			
Intervention								
No	Reference				Reference			
Yes	− 0.24	− 0.98	0.50	0.53	0.01	− 0.42	0.43	0.98

We conducted multiple sub-group analyses with respect to age, the experience of menstruation or nocturnal emission, and living place (Table 3). We did not find statistical significance in means between the intervention and the control groups.

**Table 3:** Multiple regression on reproductive health attitude and behavior in subgroups

		Coeficience	95% CI		Pr( >|t|)	Coeficience	95% CI		Pr( >|t|)
		Reproductive health attitude				Reproductive health behavior			
Age: low	Intervention								
	No	Reference				Reference			
	Yes	− 0.19	− 1.22	0.84	0.72	− 0.33	− 0.78	0.12	0.16
Age high	Intervention								
	No	Reference				Reference			
	Yes	− 0.29	− 1.33	0.75	0.59	0.57	− 0.03	1.18	0.08
Menstruation or noctornal emission: yes	Intervention								
	No	Reference				Reference			
	Yes	0.29	− 1.14	1.72	0.69	0.49	− 0.15	1.13	0.13
Menstruation or noctornal emission: no	Intervention								
	No	Reference				Reference			
	Yes	− 0.20	− 0.90	0.50	0.57	− 0.16	− 0.56	0.24	0.43
Living place: town	Intervention								
	No	Reference				Reference			
	Yes	− 0.55	− 1.36	0.25	0.18	− 0.01	− 0.39	0.37	0.95
Living place: rural	Intervention								
	No	Reference				Reference			
	Yes	0.29	− 1.02	1.61	0.66	0.04	− 0.81	0.88	0.93

## Discussion

In this quasi-experimental study, a statistically significant effect of reproductive health education on adolescent health attitudes and behaviors one year after the intervention in the multiple regression analyses was not found. To the best of our knowledge, this is one of the few studies that provided reproductive health education for adolescents in rural Tanzania, and that evaluated its long-term effects using a large sample.

Compared to other studies on similar research topics, two clinical trials of sex educational intervention and reproductive health lesson materials in urban Tanzania showed beneficial effects on adolescents' skills for safe sexual behavior and significantly decreased their unsafe sexual behaviors [29]. Another quasi-experimental study in urban Tanzania also found that a reproductive health awareness program, using the same program as this study, improved adolescents’ knowledge and behavior regarding sexuality and decision-making [26]. The differences in our findings from those could possibly be explained by the distinct research setting: the rural areas in this study. Even though there were schools in town, the academic level of students and economic environments differed considerably from those in the largest city of Dar es Salaam. In resource-limited Tanzanian rural areas, the major determinants of improvements in the reproductive health of adolescents remain at the community or structural level, including poverty and economic stability, health service provision (e.g., contraceptive services for adolescents), and support for gender equality [13, 30–32]. As a result, the effects of the sex education program could be small and offset by those determinants [33].

The content of adolescent health education can influence this effect. Although the same program previously had significant effects [24], this study involved primary and secondary schools. The increase in the content, especially for secondary students, was discussed; however, it was decided to teach decision-making mainly to avoid unwanted pregnancy, and this could not expand more on sex education, that is, use of protection such as modern contraceptive, because of the opposition of the local collaborators. Similarly, a review study reported a preference for abstinence education in Tanzania and the restrictions concerning reproductive health education and family planning taught by NGOs [7]. Based on these findings, peer education strategies were used in secondary schools, and students taught one another according to our qualitative study published earlier [34]. It was important to continue finding a better solution so that the contents are accepted in the context and are effective at the same time.

Another possible explanation for the null effects of the reproductive health education intervention on adolescents’ attitudes and behaviors could be the period of follow-up in this study. Although the effect was not observed in the period of one year, the real effects (i.e., long-term effect) of reproductive health education could have been censored given the younger age of participants who received the intervention. Thus, we may be unable to observe its effects until participants reach an older age, given that 80% of the participants in this study who received the reproductive health education interventions, were primary school students. For instance, primary studies reported that adolescents and young persons are more likely to “engage in sexual activities with older men to get money” [35]. In this case, there would be no improvements in such behavior, but there is a chance that the intervention may have reduced the risk of implementing this behavior in the long run [30]. Future studies with a long follow-up period are warranted to determine long-term effects.

This study could not rule out the possibility that the observed null effect could be due to residual confounding effects by unmeasured risk factors, such as parents' factors (education, attitudes), household income, teachers' factors, etc. A possible explanation is that the scores of the outcome measurements used in this study decreased by a certain age and increased at a later age, and it was difficult to capture the mean differences. From the figures, it is clear that the movement of the scores is not linear. Three group analyses were conducted according to age, but significant differences in the outcome measures were not found.

These findings leave many unanswered questions. First, what are the potentially unknown factors that influence the scores? One potential factor is the parental factor. This study asked if they talked with parents in general, and, specifically, about sexual issues, but parental factors were not measured, as parents were not included as participants. A systematic review and meta-analysis of parental factors associated with depression and anxiety among adolescents showed parental factors, such as less warmth, more inter-parental conflict, over-involvement, and aversiveness, and for depression, they also included less autonomy being granted and monitoring, which were associated with depression and anxiety [36]. Another systematic review of adolescent-parent communication on sexual and reproductive health issues reported that being an urban dweller, being knowledgeable, and agreeing on the importance of discussion were significantly associated with the adolescent-parent communication [37]. Therefore, the target of adolescent girls and boys in rural areas needs support for adolescent-parent communication.

Moreover, there is also a need for healthcare professionals to provide school-based sexual and reproductive health education to adolescents. This is because of the notion that sexual health education is culturally taboo in socially conservative Tanzania; both health professionals and parents do not talk about sex with adolescents [38, 39]. Hence, it is important to consider measures concerning health professionals and parental factors for future studies. In addition, education can also be expanded to parents if they have less knowledge of sexual reproductive health and gender issues in society.

## Conclusion

In this quasi-experimental study, a statistically significant effect of reproductive health education on adolescent health attitudes and behaviors one year after the intervention in multiple regression analyses could not be found. However, other studies suggest that reproductive health education intervention has improved attitudes and behaviors of adolescent students toward preventing unintended pregnancy. We could follow the children up for long-term effects and include measurements of parental factors for the next study. The project can be expanded to include parents so that parents could learn about reproductive health, and the contents could also be updated with parental perceptions.

## Data Availability

The datasets used and analyzed during this study are available from the corresponding author upon reasonable request.

## Abbreviations

JICA: Japan International Cooperation Agency
SD: Standard deviation
CI: Confidence interval
WHO: World health organization
NPO: Non-Profit Organization
NGO: Non-Governmental Organization
NIMR: National Institute for Medical Research
